# Determination of a Threshold Dose to Reduce or Eliminate CdTe-Induced Toxicity in L929 Cells by Controlling the Exposure Dose

**DOI:** 10.1371/journal.pone.0059359

**Published:** 2013-04-05

**Authors:** Xiaorun Liu, Meng Tang, Ting Zhang, Yuanyuan Hu, Shanshan Zhang, Lu Kong, Yuying Xue

**Affiliations:** 1 Key Laboratory of Environmental Medicine and Engineering, Ministry of Education, School of Public Health, Southeast University, Nanjing, China; 2 Jiangsu Key Laboratory for Biomaterials and Devices, Southeast University, Nanjing, China; King Abdullah University of Science and Technology, Saudi Arabia

## Abstract

With the widespread use of quantum dots (QDs), the likelihood of exposure to quantum dots has increased substantially. The application of quantum dots in numerous biomedical areas requires detailed studies on their toxicity. In this study, we aimed to determine the threshold dose which reduced or eliminated CdTe-induced toxicity in L929 cells by controlling the exposure dose. We established a cellular model of acute exposure to CdTe QDs. Cells were exposed to different concentrations of CdTe QDs (2.2 nm and 3.5 nm) followed by illustrative cytotoxicity analysis. The results showed that low concentrations of CdTe QDs (under 10 µg/mL) promoted cell viability, caused no obvious effect on the rate of cell apoptosis, intracellular calcium levels and changes in mitochondrial membrane potential, while high concentrations significantly inhibited cell viability. In addition, reactive oxygen species in the 10 µg/mL-treated group was significantly reduced compared with the control group. In summary, the cytotoxicity of CdTe QDs on L929 cell is dose-dependent, time-dependent and size-dependent. Low concentrations of CdTe QDs (below 10 µg/mL) may be nontoxic and safe in L929 cells, whereas high concentrations (above 10 µg/mL) may be toxic resulting in inhibition of proliferation and induction of apoptosis in L929 cells.

## Background

Quantum dots (QDs) are semiconductor nanocrystals with a diameter ranging from 2 to 100 nm and are composed of groups II–VI or III–V elements [1] which have the advantages of a broad excitation spectrum, high extinction coefficients and a narrow emission spectrum [2], [3], [4], [5]. QDs have superior optical properties over other fluorescent dyes and fluorescent nanoparticles due to their photostability, long fluorescent lifetime and high resistance to photobleaching [6], [7], [8], [9], thus, CdTe QDs are gaining increasing attention for potential use in biomedical applications such as bioimaging of tissues, disease diagnosis and biological labeling due to their unique optical and electronic properties [10], [11], [12], [13]. With the wide application of CdTe QDs, exposure to these particles in humans and the environment has raised significant concern with regard to their unanticipated and potentially undesirable harmful side effects. If CdTe QDs are used under safe conditions and are expected to play an important role in the national economy and biomedical fields, it is important to understand the toxic effects of these particles and develop screening methods to eliminate or reduce these effects.

Cell-based in vitro studies play an essential role in meaningful toxicity testing. Numerous toxi- cological assays have been carried out in order to investigate QD cytotoxicity [14], [15], [16], . Some notable results have been reported regarding the potential cytotoxicity of QDs and related mechanism [18], [19], [20], [21]. Previous studies have shown that apoptosis is the major route of cell death in the case of cadmium cytotoxicity [22], [23]. It has also been suggested that induction of apoptosis is involved in cytotoxicity. It is certain that mechanisms of toxicity of many types of nanoparticles are oxidative. Dumas [24] found that significant amounts of hydroxyl radicals were produced from the QD solutions. Although CdTe is not energetically likely to directly oxidize water to generate OH radicals, indirect mechanisms such as peroxide photolysis/photocatalysis can occur in aqueous solution. Lovric [25] found that QDs could generate reactive oxygen species (ROS), such as hydrogen peroxide and various hydroperoxide radicals, which impaired the plasma membrane, mitochondria and the nucleus, leading to severe cell dysfunction, and even cell death. Another group found that QDs could trigger oxidative processes involving singlet oxygen or electron transfer from QDs to oxygen which induced subsequent lysosomal enlargement and intracellular redistribution. Intracellular calcium levels play an important role in the development of ROS injury, one deleterious consequence due to ROS exposure is the occurrence of large increases in intracellular calcium levels [26], [27]. Considering that QDs can generate ROS and induce oxidative stress, the interference by QDs on calcium signals could be a possible mechanism of QD toxicity. Mitochondria is not only a cellular organelle, but participate in various intracellular processes including cellular Ca^2+^ signaling. They can modulate the amplitude and spatiotemporal organization of cytoplasmic Ca^2+^ signals due to their ability to rapidly accumulate and release Ca^2+^ into the cytosol [28]. Mitochondrial Ca^2+^ overload leads to ROS overproduction, which in turn triggers MPTP (mitochondrial permeability transition pore) opening and apoptotic mechanisms [29], [30]. Researchers have demonstrated the toxicity of QDs on the basis of several significant parameters, and previous reports have mostly focused on their physicochemical characteristics [31], size, shape, outer coating bioactivity [32], [33], [34], cell type and exposure time, and little attention has been paid to the exposure concentration. If QDs are used under a certain threshold concentration, they can be innocuous. Some reports have also indicated a dose-dependent cytotoxicity [35], however, limiting QD toxicology by controlling their concentration is still poorly understood due to the paucity of QD toxicological investigations.

In this study, we determined the threshold concentration which reduced or eliminated CdTe-induced toxicity in L929 cells by controlling the exposure dose to provide a valuable reference for a safe dose range. L929 cells were used in this study as an in vitro model, which is widely used in toxicology. Firstly, L929 cells were treated with a concentration range of CdTe QDs for various times. The low-concentration promoting and high-concentration inhibiting effects of CdTe QDs on cell viability were tested. Secondly, the effects of CdTe QDs exposure in L929 cells were comprehensively evaluated through the analysis of cell apoptosis, intracellular ROS production, intracellular calcium levels and changes in mitochondrial membrane potential to explore the potential toxic mechanism.

## Materials and Methods

### Quantum Dot Synthesis

The Quantum Dots (QDs) used in this study were 2.2 nm and 3.5 nm in size and synthesized by the Department of Biomedical Engineering, Southeast University, China. Briefly, the generation of CdTe precursors was obtained using an applied potential of −1.2 V in the electrolyte containing 2.0 mmol/L CdCl_2_ and 16.6 µL 3-mercaptopropionic acid (MPA) at pH 10 adjusted with 0.1 mol/L NaOH. The solution of CdTe precursors was then heated in a water bath with moderate stirring at 80°C. The CdTe QDs were gradually crystallized and their size was controlled by changing the heating time to 2 h and 20 h to obtain 2.2 and 3.5 nm-sized CdTe QDs. The resulting CdTe QDs were deposited by acetone, and after centrifugation the precipitate was washed with acetone at least three times and re-dissolved in water. More details are available in a previously published report [36].

### Cell Culture

L929 cells (mouse fibroblasts) were purchased from Shanghai Institute of Cell Biology, Chinese Academy Sciences. L929 cells were one of the first and most widely used cells in cytotoxicity test. Since it has the advantages of easy to culture and subculture in vitro, breed rapidly and easy to store, it was applied as cell lines of cytotoxicity evaluation for many materials. L929 cells were recommended as the standard cell line in the cytotoxicity test by American Society for Quality Control in 1982 [37]. L929 cells used in the cytotoxicity tests were cultured in RPMI-1640 medium supplemented with 10% fetal bovine serum, 100 U/mL penicillin and 100 µg/mL streptomycin (Gibco, USA), and cultured at 37°C in a humidified atmosphere with 5% CO_2_ and 95% air. Cells were then treated with fresh culture medium containing various concentrations of CdTe. When Cells increased doubled and redoubled, the vitality of cells was the best, this phase can be identified as the logarithmic growth phase cells, cell in the logarithmic growth phase were used in all the experiments. All the treatments were performed in triplicate in three independent experiments.

### MTT Assay

Percentage cell survival was measured using the (3-[4,5-dimethylthiazol-2-yl]-2,5- diphenyltetrazolium bromide) (MTT) colorimetric assay. L929 cells were grown until they reached 80% confluence, they were then plated into 96-well culture plates at a density of 8000 cells/well in a total volume of 100 µL and allowed to attach and grow for 24 h. The supernatant in each well was then replaced with 1640 medium containing various concentrations of QDs: 0, 2.5, 5, 10, 20, 40, 80, 120, 180 and 240 µg/mL. After 12, 24, and 48 h of incubation, 100 µL of MTT (Sigma-Aldrich, Shanghai, China) was added to each well. After 4 h incubation, the supernatant was removed and 150 µL dimethyl sulfoxide (Sigma-Aldrich, Shanghai, China) was added to each well. Samples were then shaken for 15 min to dissolve the dark blue crystals. Spectrophotometric data were measured using an automatic microplate reader (Dynex Technologies Company, USA, type MRX) at a wavelength of 490 nm. All experiments were performed in triplicate.

### Morphological Changes

L929 cells were plated in a six-well culture plate at a density of 1×10^5^ cells/mL and treated with 10, 20 and 40 µg/mL of QDs for 24 h. The dishes were then washed twice with phosphate- buffered saline (PBS) to remove the culture medium, and the cells were double stained with a mixture of Hoechst 33342 (Sigma-Aldrich, Shanghai, China) and propidium iodide (PI) (Sigma-Aldrich, Shanghai, China). The dyes were added at a final concentration of 5 µg/mL and 10 µg/mL, respectively. Cells were observed using a fluorescence microscope after incubation at 37°C for 15 min. Normal cells were stained blue by Hoechst 33342, cell morphology was healthy and the nucleus was big, while apoptotic cells showed characteristics of irregular cell outline, chromatin condensation and nuclear membrane disruption, necrotic cells were stained red by PI.

### Annexin V-FITC/propidium Iodide Apoptosis Assay

Normal, apoptotic, and necrotic cells were distinguished using an Annexin V-FITC/propidium iodide assay kit (KeyGEN Biotech, Nanjing, China) according to the manufacturer’s instructions. L929 cells were plated in a six-well culture plate at a density of 1×10^5^ cells/mL and treated with 10, 20 and 40 µg/mL of QDs for 24 h. Thereafter, cells were harvested and washed with PBS, resuspended in 400 µL of binding buffer to a density of 1×10^6^ cells/mL, and 5 µL of Annexin V-FITC was then added to the samples. After incubation for 15 min at 4°C in the dark, 10 µL of propidium iodide was added and the cells were incubated for 5 min. Flow cytometry (BD FACSCalibur) analysis was performed within 15 min.

### Determination of Intracellular Reactive Oxygen Species (ROS) Generation

The level of intracellular ROS was analyzed using 2′,7′-dichlorofluorescin diacetate (DCFH-DA) (Sigma, MO, USA), which is an oxidation-sensitive fluorescence probe. DCFH-DA upon enzymatic hydrolysis by intracellular esterases forms non-fluorescent DCF-H, which is subsequently oxidized to highly fluorescent DCF in the presence of ROS [38], [39]. Therefore, the degree of DCF fluorescence intensity demonstrates the amount of ROS formed in the cells. After treatment with 10, 20, and 40 µg/mL of QDs for 24 h, the cells were rinsed twice with PBS and then loaded with DCFH-DA 10 µM diluted in serum-free medium and incubated at 37°C for 30 min. All samples were rinsed three times with serum-free medium, and then resuspended in PBS. Fluorescence was measured using flow cytometry at excitation and emission wavelengths of 488 and 525 nm for DCF fluorescence within 30 min.

### Measurement of Intracellular Calcium Levels ([Ca^2+^]i)

Changes in [Ca^2+^]_i_ were measured with the fluorescent probe fluo-3/AM. Fluorescence intensity after labeling was measured using confocal microscopy. For confocal microscopic analyses, the cells were cultured in six-well plates at a density of 1×10^5^ cells/well and treated with 10, 20 and 40 µg/mL of QDs for 24 h. The dishes were then washed twice with PBS to remove the culture medium. Treated and untreated L929 cells were loaded with 10 µM fluo-3/AM incubated at 37°C for 30 min and then washed with PBS containing calcium to remove excess fluo-3/AM before imaging. Micrographs were captured by confocal microscopy (100 M; Carl Zeiss Meditec, GmbH, Jena, Germany) at an excitation wavelength of 488 nm and an emission wavelength of 525 nm within 30 min.

### Detection of Changes in Mitochondrial Membrane (ΔΨm)

Mitochondrial membrane potential was measured using the JC-1 Apoptosis Detection Kit (KeyGEN Biotech, Nanjing, China). L929 cells were cultured at a density of 1×10^5^ cells/well in six-well plates and treated with 10, 20 and 40 µg/mL of QDs for 24 h. The cells were then collected, centrifuged, and rinsed twice with PBS, resuspended in 500 µL 1× Incubation Buffer including 1 µL JC-1 loaded for 30 min at 37°C in the dark. All samples were then rinsed twice with 1×Incubation Buffer and analyzed immediately by flow cytometry at an excitation wavelength of 488 nm and an emission wavelength of 530 nm.

### Statistical Analysis

All experiments were carried out at least three times unless otherwise indicated. Data are presented as the mean ± standard deviation (SD). Statistical significance was tested among and between groups using one-way analysis of variance followed by Dunnett’s post hoc test. The results were considered significant if *p<0.05*, when compared to the control.

## Results

### QD Preparation and Characterization

The water-soluble MPA-capped CdTe QDs were prepared according to published procedures. The size and morphology of the CdTe QDs were characterized with high-resolution transmission electron microscopy (HR-TEM), Our TEM study illustrates that the average size of CdTe QDs were 2.2±0.25 nm and 3.5±0.49 nm at heating time of 2 and 20 h (Figure S1). The absorption peaks occur at 514 and 578 nm, and the PL emission peaks are at 547 and 622 nm (λex  = 388 nm) for the heating times of 2 and 20 h, respectively (Figure S2). The fluorescent color under UV irradiation changed from green to red with increasing heating time. Comparable dimensions (9.82±1.14 nm and 7.39±0.74 nm for 3.5 nm and 2.2 nm CdTe QDs) in water solution, and (26.79±1.59 nm and 15.65±1.63 nm for 3.5 nm and 2.2 nm CdTe QDs) in cell culture media (RPMI-1640 supplemented with 10% fetal bovine serum) after 24 h incubation were confirmed by dynamic light scattering (DLS). The surface charge through ζ-potential measurements were (−26.46±4.75 mV and −31.84±3.06 mV for 3.5 nm and 2.2 nm CdTe QDs) in water solution, and (−13.86±3.44 mV and −15.73±4.10 mV for 3.5 nm and 2.2 nm CdTe QDs) in cell culture media (RPMI-1640 supplemented with 10% fetal bovine serum) after 24 h incubation (Figure S3). Our results show that the size of both CdTe QDs confirmed by DLS in cell culture media are greater than in water solution, and the ζ-potential of both CdTe QDs in cell culture mediain are lower than in water solution. This maybe reflects the formation of a protein corona around the QDs, as already observed for other kinds of nanoparticles [40], [41].

### Cytotoxicity

**Figure 1:**
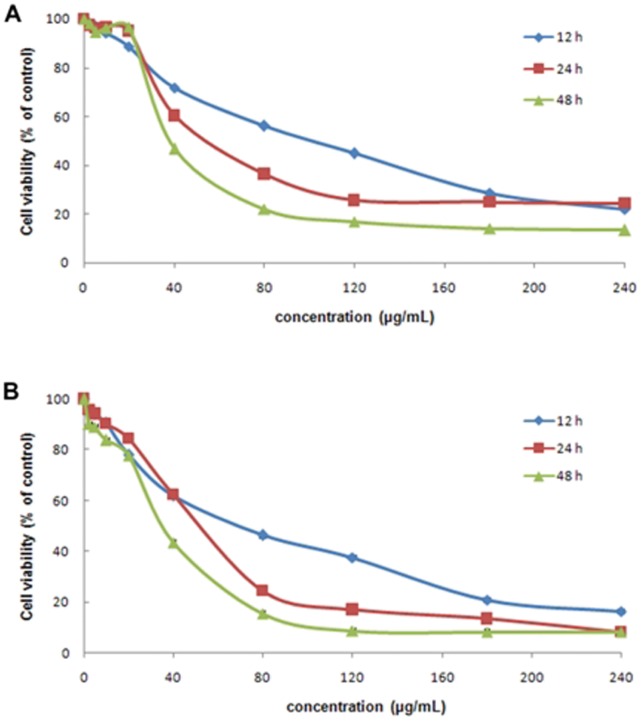
MTT assay of L929 cell viability after various treatments. L929 cells treated with (A) 3.5 nm CdTe QDs and (B) 2.2 nm at 2.5–240 µg/ml for 12, 24 and 48 h. Typical data from one of three independent experiments are shown with similar results compared to the control group.

To test the potential cytotoxicity of CdTe QDs at different concentrations, we used MTT assays to examine the viability of L929 cells and the results are shown in Figure 1. L929 cells were exposed to 3.5 nm and 2.2 nm CdTe QDs for 12, 24 and 48 h at a dosage range from 2.5 to 240 µg/mL. Both sizes of CdTe QDs had little effect on the viability of L929 cells when the concentration was below 20 µg/mL at the various periods (approximately 90% and 80%, respectively), however, when the concentration was higher than 20 µg/mL, viability decreased markedly with increased concentration and time. When the concentration was above 180 µg/mL, cell viability decreased to less than 20%. At the same level of dose and time, the viability of L929 cells was lower when exposed to 2.2 nm CdTe QDs than to 3.5 nm CdTe QDs, the 2.2 nm particles showed much higher cytotoxicity than the 3.5 nm particles.

### Apoptosis

**Figure 2:**
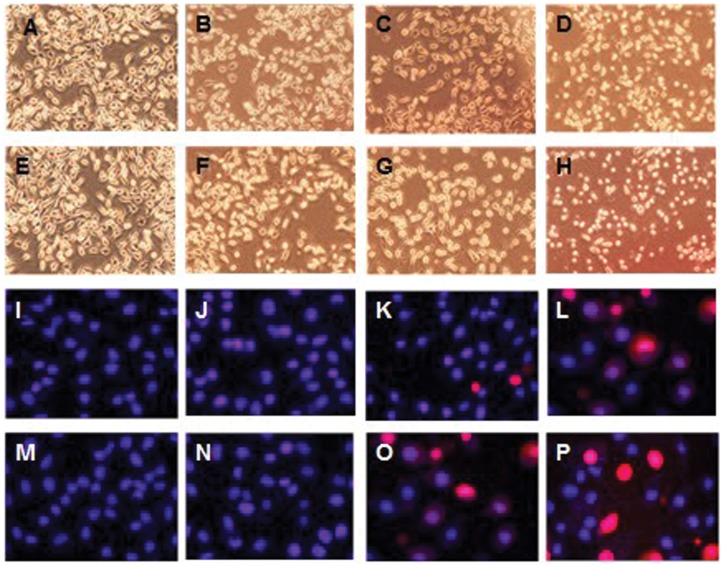
Representative fluorescent images showing morphological changes in L929 cells treated with CdTe QDs. (A,I)-control treatment, (B,J) −3.5 nm CdTe 10 µg/ml treatment, (C,K) −3.5 nm CdTe 20 µg/ml treatment, (D,L) −3.5 nm CdTe 40 µg/ml treatment; (E,M)-control treatment, (F,N) −2.2 nm CdTe 10 µg/ml treatment, (G,O) −2.2 nm CdTe 20 µg/ml treatment, (H,P) −2.2 nm CdTe 40 µg/ml treatment.

To investigate the influence of different concentrations of CdTe QDs on apoptosis, morphological changes in L929 cells were observed by fluorescence microscopy after 24 h of exposure to 3.5 nm and 2.2 nm CdTe QDs (Figure 2A–D and E–H). Untreated cells (controls) showed healthy morphology. Cells exposed to 3.5 nm and 2.2 nm CdTe QDs did not show significant changes compared to the control cells at the concentration of 10 µg/mL, however, when exposed to 20 µg/mL, cells exhibited similar morphological impairment and showed typical features of the early stages of apoptosis including cell shrinkage, membrane fragmentation, irregularity of cell outline, cell detachment due to loss of adhesion, and oval-shaped appearance (Figure 2C, Figure 2G). In contrast, cells treated with 40 µg/mL displayed characteristics of the late stages of apoptosis which included the formation of apoptotic bodies and secondary necrotic cells (Figure 2D, Figure 2H). We stained L929 cells with Hoechst 33342/PI to observe the morphological changes in more detail exposure to 3.5 nm and 2.2 nm CdTe QDs (Figure 2 I–L and M–P). It can be seen that untreated cells were round and blue in color, while cells treated with a high concentration were necrotic, apoptotic and red in color. In contrast, treated apoptotic cells showed brighter blue light emission than untreated cells.

**Figure 3:**
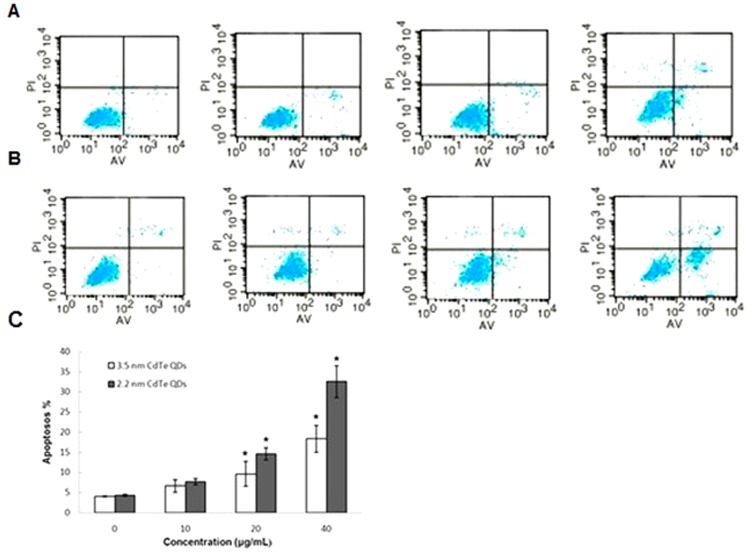
Annexin V-FITC/PI double staining analysis of apoptosis in L929 cells (24 h). **A**: L929 cells treated with 3.5 nm CdTe QDs at 0, 10, 20 and 40 µg/ml for 24 h. **B**: L929 cells treated with 2.2 nm CdTe QDs at 0, 10, 20 and 40 µg/ml for 24 h. Top right quadrant, dead cells in the late stage of apoptosis; bottom right quadrant, cells undergoing apoptosis; bottom left quadrant, viable cells. **C**: Quantitative analysis of apoptotic cells after the treatments shown in A and B. Results represent the mean ± SD of three independent experiments. One-way analysis of variance followed by Dunnett’s post hoc test was used for statistical analysis. *p<0.05, indicates a statistically significant difference when compared to the control.

To quantify cell apoptosis further, the L929 cells exposed to 2.2 nm and 3.5 nm CdTe QDs at concentrations of 10, 20 and 40 µg/mL were stained using Annexin V-FITC/propidium iodide double-staining and analyzed by flow cytometry. Apoptotic cells which lose asymmetry of membrane phospholipids produce phosphatidylserine on the outer leaflet of the plasma membrane. Annexin V, a calcium-dependent phospholipid-binding protein with a high affinity for phosphatidylserine, can therefore be used as a sensitive probe to detect the presence of phosphatidylserine on the cell membrane and hence as a marker of apoptosis. Propidium iodide is a nonspecific DNA intercalating agent, which is excluded by the plasma membrane of living cells, and thus can be used to distinguish apoptotic cells from necrotic and living cells by supravital staining without prior permeabilization [42]. The results of these studies are shown in Figure 3. The percentage of apoptotic cells was 4.03%, 6.61%, 9.61% and 18.32% following treatment with 0, 10, 20 and 40 µg/mL of 3.5 nm CdTe QDs, respectively. For 2.2 nm CdTe QDs, the percentage of apoptotic cells was 4.3%, 7.68%, 14.61% and 32.52%, respectively. From these results, exposure to a low concentration (10 µg/mL) of 3.5 nm or 2.2 nm CdTe QDs had no obvious effect on cell apoptosis, and no statistically significant differences were observed when compared to the control group. However, concentrations greater than 20 µg/mL induced a much higher rate of cell apoptosis, and statistically significant differences were observed when compared to the control group. In addition, 2.2 nm CdTe QDs induced a higher rate of apoptosis in L929 cells than 3.5 nm CdTe QDs at all concentrations.

### ROS Determination

**Figure 4:**
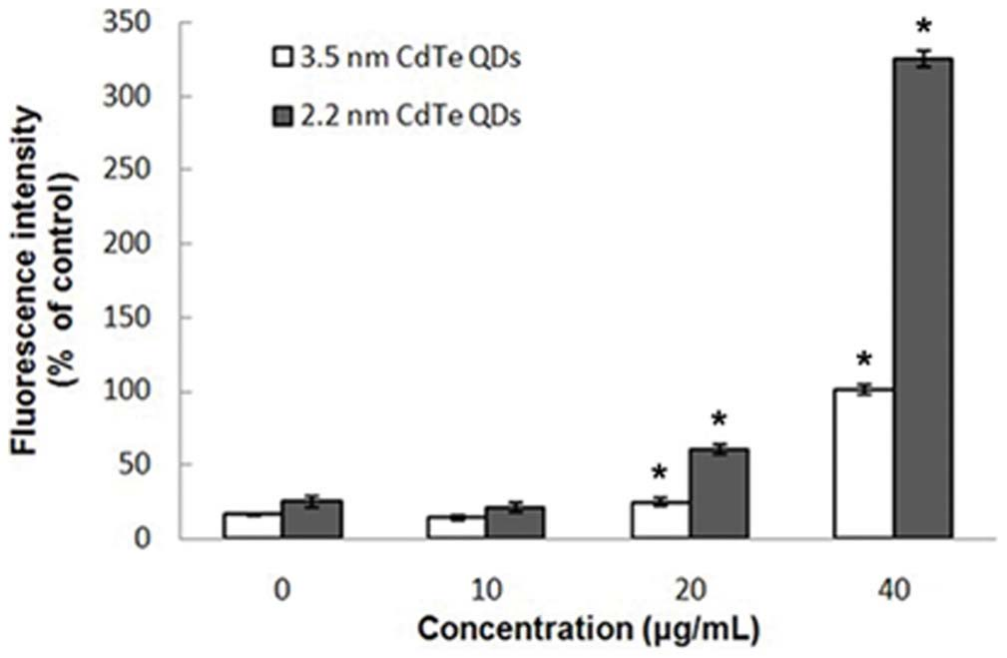
Quantification of fluorescence intensity showing the relative amount of intracellular ROS formation. ROS formation in L929 cells exposed to 0, 10, 20 and 40 µg/ml of 3.5 nm and 2.2 nm CdTe QDs for 24 h. The results were quantitatively analyzed for changes in fluorescence intensities within cells and expressed as percent units of DCF fluorescence of the control. Results represent the mean ± SD of three independent experiments. One-way analysis of variance followed by Dunnett’s post hoc test was used for statistical analysis. *p<0.05, indicates a statistically significant difference compared with the control.

Levels of ROS serve as reliable indicators of oxidative stress. The intracellular formation of ROS is believed to be the causal factor in Cd-induced cytotoxicity, therefore we used the DCFH-DA detection reagent to examine ROS generation in CdTe-treated L929 cells. As hydrogen peroxide or low-molecular weight hydroperoxides produced by cells can oxidize DCFH to the highly fluorescent compound DCF, the fluorescence intensity is thus proportional to the amount of peroxide produced by the cells [36]. As shown in Figure 4, the DCF fluorescence intensities were significantly increased following exposure to 20 and 40 µg/mL of 2.2 nm and 3.5 nm CdTe QDs (60.42±3.53 and 324.88±5.74, 24.54±2.38 and 100.95±3.54, respectively) compared to the control group (24.83±3.77 and 16.24±0.50), indicating higher amounts of ROS formation in the cells. There were no significant differences in DCF fluorescence intensities between the 10 µg/mL 2.2 nm and 3.5 nm CdTe-exposed groups (20.77±3.41, 13.92±1.51) and the control group. In addition, our results revealed that the intracellular ROS content was significantly higher in 2.2 nm CdTe treated cells than in 3.5 nm CdTe treated cells.

### Intracellular Calcium Levels

**Figure 5:**
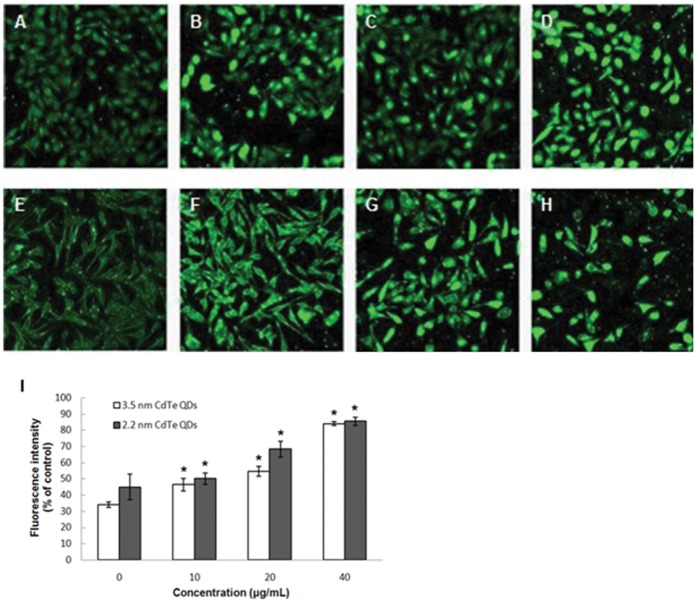
Confocal microscopic images which show the fluorescence intensity of intracellular calcium formation. A–D : L929 cells treated with 3.5 nm CdTe QDs at 0, 10, 20 and 40 µg/ml (24 h). **E–H**: L929 cells treated with 2.2 nm CdTe QDs at 0, 10, 20 and 40 µg/ml for 24 h. **I**: Quantification of fluorescence intensity. Results represent the mean ± SD of three independent experiments. One-way analysis of variance followed by Dunnett’s post hoc test was used for statistical analysis. *p<0.05, indicates statistically significant difference compared with the control.

Intracellular calcium levels were determined using the fluorescence Ca^2+^ indicator, fluo-3/AM, and analyzed by confocal microscopy. The confocal images are shown in Figure 5, and from these images it can be seen that the fluorescence intensity was dose-dependent. QDs elevated intracellular calcium levels. As shown in the 20 and 40 µg/mL 3.5 nm CdTe treatments, the fluorescence intensity increased to 54.65±3.11 and 83.98±1.22, respectively, and was significantly different to the control group (34.03±1.73). The 10 µg/mL treatment failed to induce a significant rise in intracellular calcium level compared with that in the control as the fluorescence intensity increased to 46.35±3.99. In the 20 and 40 µg/mL 2.2 nm CdTe treatments, the fluorescence intensities increased to 68.29±4.94 and 85.44±2.61, respectively, which were significantly different to that of the control group (44.95±8.03). In addition, the 10 µg/mL treatment failed to induce a significant increase in intracellular calcium level compared with the control as the fluorescence intensity changed to 50.02±3.58. These data suggest that the acute application of CdTe QDs can affect steady-state intracellular calcium by elevating intracellular calcium concentration. However, cells treated with 10 µg/mL showed little change in intracellular calcium levels.

### Mitochondrial Membrane Potential (ΔΨm)

**Figure 6:**
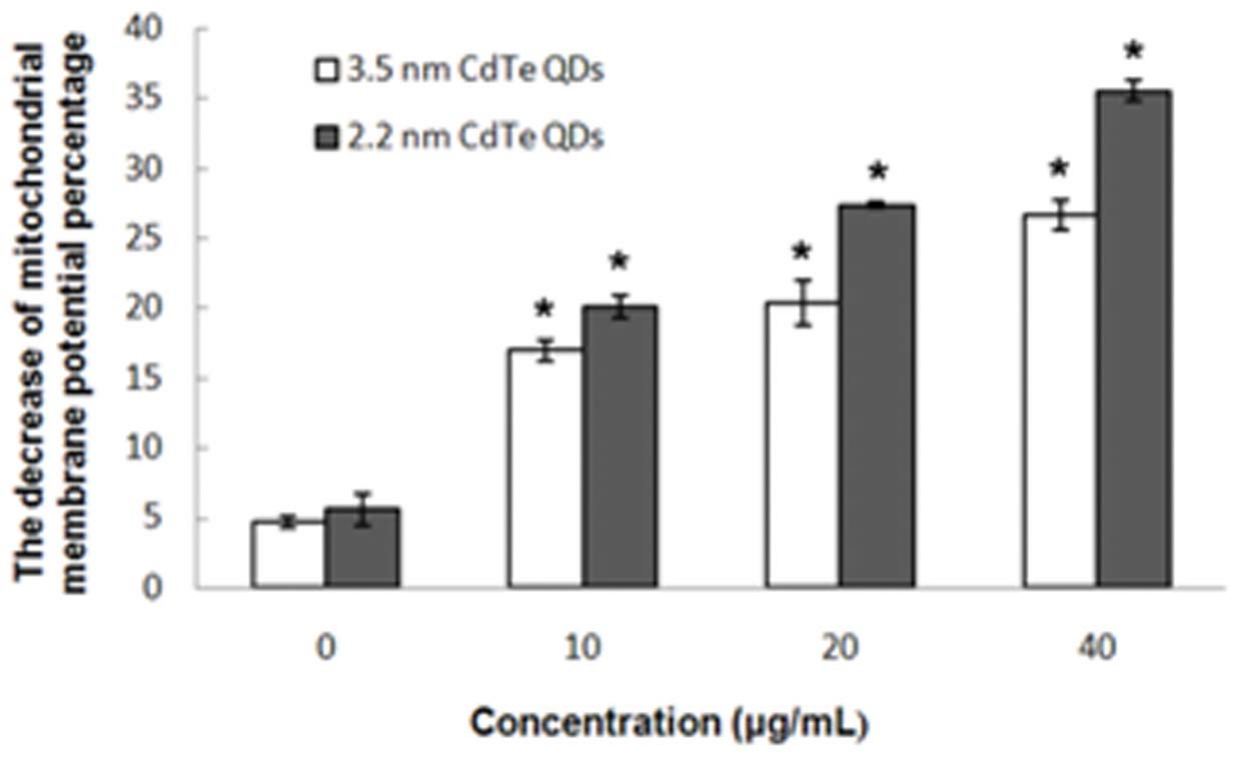
Flow cytometry demonstrated changes in mitochondrial membrane potential. L929 cells treated with 3.5 nm and 2.2 nm CdTe QDs at 0, 10, 20 and 40 µg/ml for 24 h. Mitochondria membrane potential was measured using the JC-1 Apoptosis Detection Kit. Results represent the mean ± SD of three independent experiments. One-way analysis of variance followed by Dunnett’s post hoc test was used for statistical analysis. *p<0.05, indicates statistically significant difference compared with the control.

We examined the effects of CdTe QDs on mitochondrial membrane potential as a measure of intrinsic apoptosis using JC-1, a dye which is selectively permeable to mitochondria and emits an altered wavelength as it aggregates in the interior of normally polarized mitochondria. As shown in Figure 6, our results show that treatment of L929 cells with 2.2 nm and 3.5 nm CdTe QDs led to significant losses in mitochondrial membrane potential, the percentage decrease in mitochondrial membrane potential was 4.67%, 16.98%, 20.31% and 26.66% at 0, 10, 20 and 40 µg/mL of 3.5 nm CdTe QDs, respectively. For 2.2 nm CdTe QDs, the decrease in mitochondrial membrane potential was 5.60%, 20.07%, 27.35% and 35.52%, respectively. A dose-dependent relationship was demonstrated, and statistically significant differences between the control and treated groups were observed.

## Discussion

With the development of nanotechnology science, the application of QDs in the industrial and biomedical fields will dramatically increase, as will the likelihood of exposure. Many areas of these nanoparticles are unexplored, such as their potential adverse effects on human health. With their increased application, the in vitro and in vivo biodistribution and biosafety of QDs have attracted more and more attention in recent years [43]. Some scientists have attempted to reduce the toxicity of QDs by surface modifications for further applications in biomedical science, including conjugation and capping with biomolecules and polymers [44], [45], [46]. The improved QDs may seem innocuous initially, but their accumulation in tissues or cells and long-term exposure to the bioenvironment can destabilize them, which may yield unprotected QDs. Unfortunately, unprotected QDs can impair cell structures and functions and even induce cell death [47]. Impairment of cell structures and functions and a decrease in cell viability by QDs have been observed in a large number of in vitro studies in various cell lines. To date, the available data on the toxicity of QDs have mainly been obtained from studies using high doses. However, limiting QD toxicity by controlling the concentration is still poorly understood due to the paucity of QD toxicological investigations. When the exposure dose is under a certain threshold, QDs can be non-toxic or harmless, therefore, if we want to reduce QD-induced toxicity to allow extensive use in biological labeling or imaging, we should develop non-toxic QDs by controlling the applied concentration to a low level. This may be the most effective means of producing non-toxic QDs.

Data obtained from our in vitro MTT research showed that 3.5 nm and 2.2 nm CdTe QDs both exhibited slight cytotoxicity in L929 cells at 20 µg/mL. The viability of L929 cells was approximately 90% and 80%, respectively. Our data also showed that both sizes of CdTe QDs induced sharp growth inhibition of L929 cells when the exposure concentration was above 40 µg/mL. These results also showed that the cytotoxicity of CdTe QDs was dose-dependent, time-dependent and size-dependent, which is consistent with a trend reported previously [48], [49]. Since the viability effects of high concentration CdTe QDs have been confirmed by many researchers, in the current study we focused mainly on the cellular effects of low concentrations and investigated the potential molecular mechanism. From the MTT results, the concentration range which resulted in low proliferation was identified and these concentrations (10, 20, 40 µg/mL) were thus used in the following experiments.

Apoptosis, or programmed cell death, is an important way of maintaining homeostasis in terms of cell division and cell death. Apoptosis is a regulated process which can be triggered by different stimuli and is mediated by a cascade of enzymes [50]. Apoptosis results in the fragmentation of cells into apoptotic bodies which are engulfed by neighboring cells and macrophages. To study the cytotoxicity initiated by CdTe QDs further, we investigated apoptosis in L929 cells. Morphological changes and Annexin V-FITC/PI staining showed distinct dose-dependent induction of apoptosis in L929 cells after 24 h of CdTe treatment. Exposure to a low concentration (10 µg/mL) of both 3.5 nm and 2.2 nm CdTe QDs had no statistically significant effect on morphological features and the rate of apoptosis compared to the control group, while concentrations greater than 20 µg/mL induced marked morphological changes and a much higher rate of apoptosis. From these results, we identified a threshold of 10 µg/mL for both 3.5 nm and 2.2 nm CdTe QDs which may be less toxic to L929 cells. In order to determine this threshold, we estimated intracellular ROS formation qualitatively. Oxidative stress is an important mechanism of CdTe-mediated cellular toxicity. The generation of free radicals, particularly ROS, is believed to be partially responsible for the cytotoxicity of QDs [51], [52]. Our results demonstrated that exposure to 20 µg/mL and 40 µg/mL of 3.5 nm and 2.2 nm CdTe QDs for 24 h significantly increased intracellular ROS generation in L929 cultures, which is consistent with the reports referred to above. While exposure to 10 µg/mL generated fewer intracellular ROS and reduced inhibition of ROS generation compared to the control group, these findings were the opposite of those observed following exposure to high concentrations. This may have contributed to hormesis. Hormesis is a dose-response phenomenon in which the opposite effects are observed at low, compared to high doses for the same measured parameter. This will result in either an inverted U-shaped or a J-shaped dose-response curve. The concept of hormesis has received considerable interest in the toxicological, pharmacological and general biomedical areas over the past few years [53], [54]. It is clear from the data reported so far that the generation of ROS is partially responsible for the cytotoxicity of QDs. Therefore, controlling the generation of ROS can effectively reduce the toxicity of CdTe QDs. In our experiment, 10 µg/mL CdTe QDs appeared to be non-toxic to L929 cells. Intracellular calcium signaling is generally considered to play a major role in physiological as well as pathological functions. Its concentration in the cellular environment changes in response to a range of signals that allow this ion to modulate cellular function. An overload of intracellular calcium concentration is also one of the main causes of cytotoxicity, and sustained elevation of intracellular calcium may impair various cell functions, for example, impair mitochondrial function and cause chromatin damage, and may eventually lead to cell death [55], [56]. As shown by confocal microscopy, our data showed that treatment with 20 and 40 µg/mL of 3.5 nm and 2.2 nm CdTe QDs resulted in a significant increase in the fluorescence intensity of intracellular calcium, whereas treatment with 10 µg/mL failed to induce a significant increase compared with that of the control group. We conclude that concentrations of CdTe QDs under 10 µg/mL have little effect on intracellular calcium level in L929 cells, in other words, at this concentration CdTe QDs are safe since overload of intracellular calcium concentration can cause cytotoxicity. Mitochondria are significant organelles in QD-induced toxicity [57]. Previous studies have shown that mitochondria act as important signaling conduits during programmed cell death and that loss of mitochondrial integrity can be promoted or inhibited by many key regulators of apoptosis [58]. In the present study, we found that both sizes of CdTe QDs induced a concentration-dependent loss in mitochondrial membrane potential when the concentration was greater than 20 µg/mL.

The overall findings in this study indicated that exposure to low concentrations of 3.5 nm and 2.2 nm CdTe QDs (under 10 µg/mL) have little effect on cell viability with no obvious cell apoptosis, reduced ROS generation, no notable effect on intracellular calcium level and little effect on mitochondrial membrane potential in L929 cells. This concentration may serve as a threshold level for these two sizes of QDs only in L929 cells, however, whether exposure to low concentrations of CdTe QDs can provide a protective role in humans remains uncertain and requires further vivo and other experimental study.

## Supporting Information

Figure S1 TEM image of MPA-capped CdTe QDs and its corresponding size distribution for 2.2 nm (a,b) and 3.5 nm (c,d).
(TIF)

Figure S2 UV–vis absorption (a) and PL spectra (b) of MPA-capped CdTe QDs (2.2 nm and 3.5 nm). The inset shows fluorescent photograph of as-synthesized CdTe QDs under UV irradiation.
(TIF)

Figure S3 Dynamic light scattering (A and B) and ζ-potential measurements (C and D) of 3.5 nm and 2.2 nm CdTe QDs. DLS values are the average of at least 10 runs each containing 15 sub- measurements. ζ-potential values are the average of at least 10 runs each containing 30 submeasurements.
(TIF)
