# Supplementary figures and images for: Determination of a Threshold Dose to Reduce or Eliminate CdTe-Induced Toxicity in L929 Cells by Controlling the Exposure Dose
Source: PLoS One. 2013 Apr 5;8(4):e59359. doi: 10.1371/journal.pone.0059359 (PMC3618428; doi:10.1371/journal.pone.0059359)

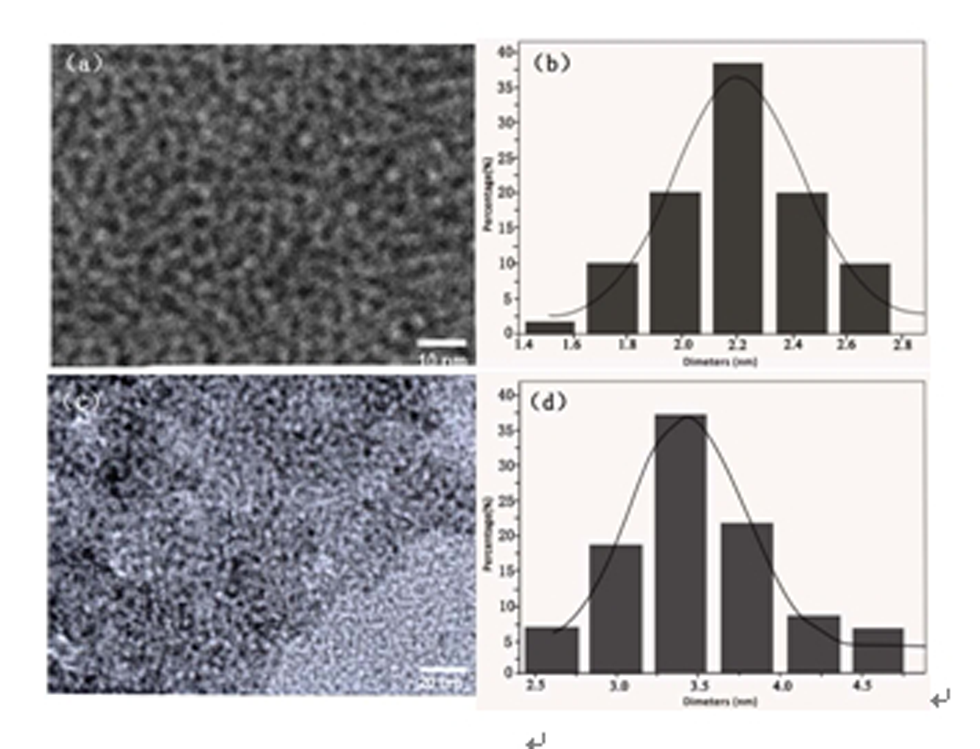

Supplement: Figure S1 — TEM image of MPA-capped CdTe QDs and its corresponding size distribution for 2.2 nm (a,b) and 3.5 nm (c,d). (TIF) [file pone.0059359.s001.tif]

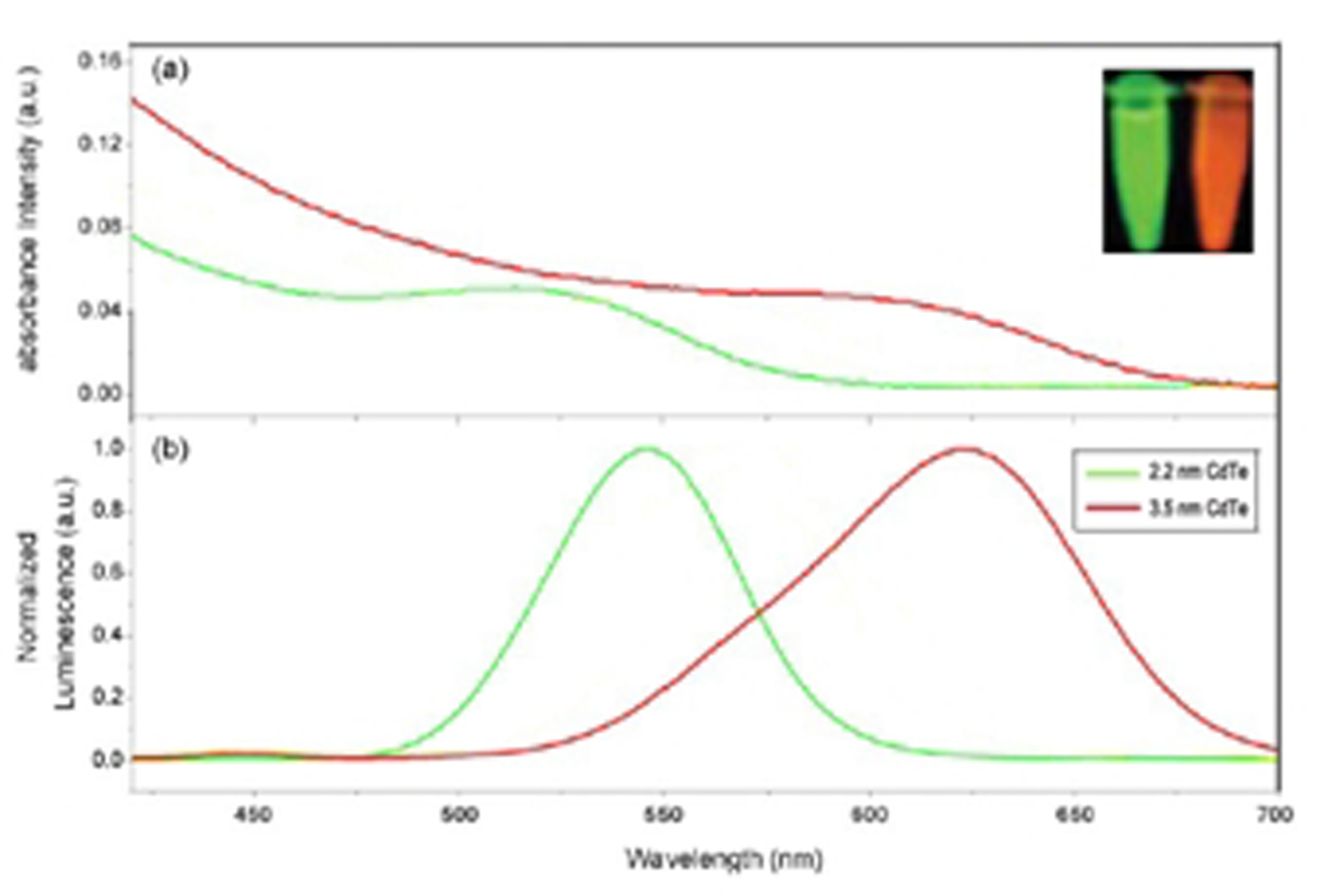

Supplement: Figure S2 — UV–vis absorption (a) and PL spectra (b) of MPA-capped CdTe QDs (2.2 nm and 3.5 nm). The inset shows fluorescent photograph of as-synthesized CdTe QDs under UV irradiation. (TIF) [file pone.0059359.s002.tif]

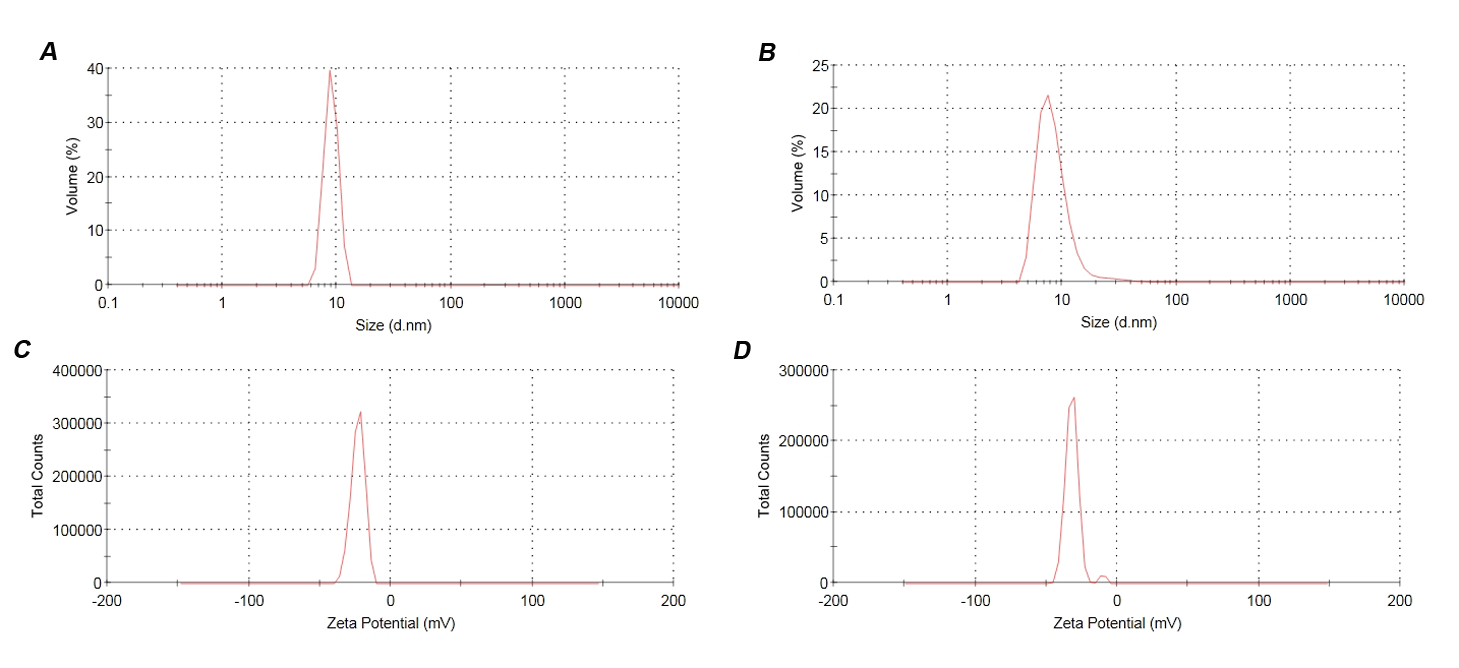

Supplement: Figure S3 — Dynamic light scattering (A and B) and ζ-potential measurements (C and D) of 3.5 nm and 2.2 nm CdTe QDs. DLS values are the average of at least 10 runs each containing 15 sub- measurements. ζ-potential values are the average of at least 10 runs each containing 30 submeasurements. (TIF) [file pone.0059359.s003.tif]
